# Nitrogen-Doped Carbon Composites Embedded with Cu/Co Species Derived from Metal-Functionalized Ionic Liquids: Design and Supercapacitive Performance

**DOI:** 10.3390/ma19173687

**Published:** 2026-08-30

**Authors:** Jiao Wu, Lingxia Liu, Liu Liu, Zhenning Zhuo, Zefeng Wei, Zhi Cui, Congxiu Guo, Jiangtao Meng

**Affiliations:** School of Electric Power, Civil Engineering and Architecture, Shanxi University, Taiyuan 030006, China; jiaow@sxu.edu.cn (J.W.); liulingxia@sxu.edu.cn (L.L.);

**Keywords:** ionic liquid, metal/carbon composites, pseudocapacitance

## Abstract

Although traditional carbon-based supercapacitor electrode materials exhibit good cycling stability and conductivity, their specific capacitance and energy density are limited by the dual-layer capacitor storage mechanism, making it difficult to satisfy the growing demand for high-performance energy storage. Consequently, the introduction of pseudocapacitive materials with Faradaic charge transfer characteristics has become a research focus in the field of supercapacitors. Herein, Cu/Co metal-anchored carbon composites (IL-Cu@NC and IL-Co@NC) have been successfully synthesized using metal-functionalized ionic liquids as both precursors and dopants via a one-step carbonization process. The micro-morphology, crystallinity, surface chemical states, and pore structure of the prepared electrode materials have been systematically characterized using scanning electron microscopy (SEM), chronoamperometry, X-ray diffraction (XRD), X-ray photoelectron spectroscopy (XPS), Raman spectroscopy, and N_2_ adsorption–desorption measurements. The energy storage performance of the fabricated electrode materials has been investigated using a three-electrode system in an alkaline solution. The optimized IL-Cu@NC electrode achieves a specific capacitance of 653 F g^−1^ at 0.5 A g^−1^, along with stable cycling performance. This research proposed a feasible strategy for developing high-performance supercapacitor electrodes by functionalizing metals with ionic liquids, achieving enhanced specific capacitance compared with conventional carbon materials.

## 1. Introduction

Supercapacitors play a significant role in the field of electrochemical energy storage due to the high power density, long-term stability, and excellent rate response [1,2,3]. Based on the charge storage mechanisms, supercapacitors can be generally categorized into double-layer capacitors (EDLCs) and pseudocapacitors. The former typically uses carbon-based materials as the active electrode material, and the energy storage process relies on the reversible electrostatic adsorption of electrolyte ions at the interface of the electrode [4,5]. Carbon materials have been widely used in EDLC research because of the high specific surface area, tunable pore structure, excellent conductivity, and chemical inertness [6]. However, limited by their surface physical adsorption capacity, EDLCs suffer from significant shortcomings in terms of specific capacitance and energy density, which pose challenges in meeting the requirements of future high-energy applications [7]. In contrast, pseudocapacitors primarily use transition metal oxides such as RuO_2_ or conductive polymers as electrode active materials [8]. The energy storage process in pseudocapacitors relies on rapid, reversible Faradaic redox reactions at the electrode surface, resulting in specific capacitance performance that is significantly superior to that of carbon materials. However, the high cost of precious metal-based materials and the limited natural reserves severely constrain their large-scale commercial application [9]. Therefore, the development of low-cost, abundant, and highly pseudocapacitive non-precious metal-based alternative materials has become a key focus and challenge in this field.

Therefore, researchers have gradually focused their attention on transition metal–carbon composite electrodes in order to achieve synergistic optimization between energy density and cost. Transition metals (such as Mn, Ni, Co, and Fe) exhibit excellent redox activity due to their multiple accessible oxidation states and partially filled d-orbitals, which enable reversible faradaic electron transfer at the electrode surface [10,11,12,13,14]. By incorporating these active metal components into the carbon matrix, this approach not only preserves the multi-level pore structure and excellent electrical conductivity of the carbon materials but also introduces an additional pseudocapacitive effect, which provides higher specific capacitance and thereby effectively addresses the limitation of traditional EDLC electrode materials in terms of energy density [15,16]. It is worth noting that the synthesis of most metal-doped carbon materials currently still relies on expensive organometallic precursors or involves complex, multi-step processes, which limits their engineering applications. Therefore, it is crucial to develop cost-effective precursors with high metal loading to achieve synergistic optimization of the controlled synthesis and electrochemical performance of metal-loaded carbon materials.

Recently, ionic liquids have been regarded as precursor materials that can serve as both carbon sources and dopants, owing to their tunable anion–cation structures, extremely low vapor pressures, high thermal stability, and good intrinsic conductivity [17,18]. Particularly, the functionalized ionic liquids containing transition metal coordination anions can introduce metal species into carbon precursors in a molecularly dispersed state, and metal/nitrogen co-doped carbon materials can then be directly obtained through a simple pyrolysis process. Therefore, it can effectively avoid problems such as metal agglomeration and uneven distribution in traditional impregnation methods or involving the external addition of metal salts [19,20].

Herein, two ionic liquids containing different transition metals ([BMIm]_2_[CuCl_4_] and [BMIm]_2_[CoCl_4_]) were synthesized using 1-butyl-3-methylimidazole chloride ([BMIm]Cl) as the cationic structural unit and CuCl_2_·2H_2_O as well as CoCl_2_·6H_2_O as the metal ligands, respectively. Subsequently, the aforementioned two ionic liquids were used simultaneously as carbon precursors and metal dopants to prepare metal-loaded carbon composites (IL-Cu@NC and IL-Co@NC) via a one-step pyrolysis process. The surface morphology, chemical composition, phase structure, pore characteristics, and electrochemical energy storage behavior of the prepared samples have been systematically characterized. The typical sample (IL-Cu@NC) exhibits remarkable specific capacitance (reaching 653 F g^−1^ at 0.5 A g^−1^) and good cycling stability.

## 2. Materials and Methods

### 2.1. Preparation of Metal-Ion-Containing Liquids

To prepare a copper-containing metal ionic liquid, the ionic liquid [BMIm]Cl was mixed with CuCl_2_·2H_2_O in a molar ratio of 2:1. First, 6.99 g (0.04 mol) of powdered [BMIm]Cl and 3.41 g (0.02 mol) of CuCl_2_·2H_2_O were weighed (both reagents were obtained from Shanghai Macklin Biochemical Technology Co., Ltd., Shanghai, China). The above reagents were transferred to a beaker and stirred for 24 h at 80 °C. Then, the product was loaded into an oven and vacuum-dried at 70 °C for 24 h, yielding a dark green, viscous liquid. The obtained liquid is the target ionic liquid, namely [BMIm]_2_[CuCl_4_].

The preparation of an ionic liquid containing cobalt metal follows the same method as that described above for preparing an ionic liquid containing copper metal. By replacing CuCl_2_·2H_2_O with CoCl_2_·6H_2_O (from Shanghai Macklin Biochemical Technology Co., Ltd., Shanghai, China), a violet-blue cobalt-containing metal ionic liquid was prepared, specifically ([BMIm]_2_[CoCl_4_]). The detailed synthesis reaction is shown in Figure S1.

### 2.2. Preparation of Metal-Containing Ionic Liquid-Derived Carbon Materials

**Figure 1:**
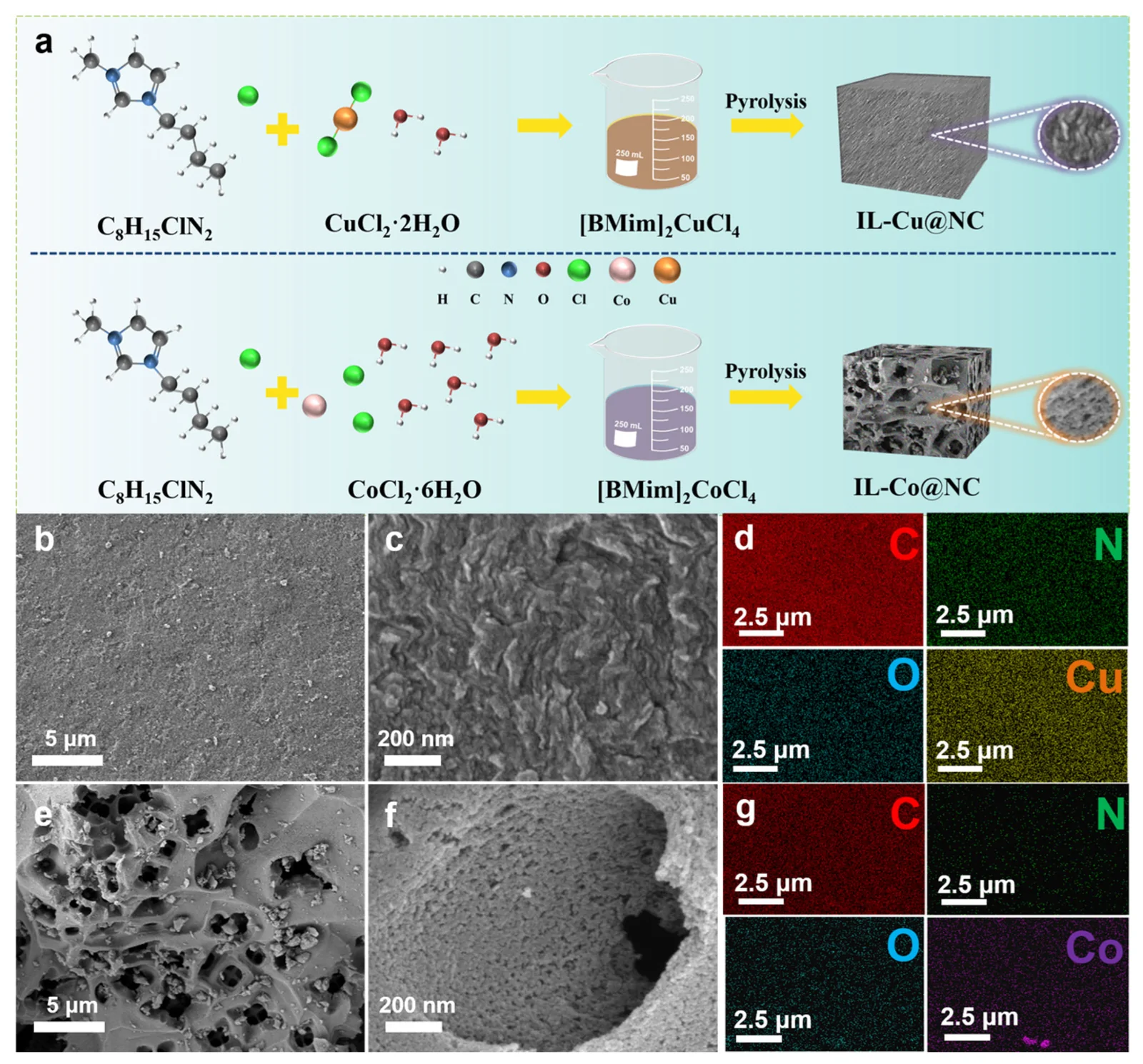
Schematic diagram of synthesis of metal-ion-liquid-derived carbon materials (**a**); SEM images of IL-Cu@NC (**b**,**c**); IL-Co@NC (**e**,**f**); and the EDS spectra of C, N, O and Cu in IL-Cu@NC (**d**) and of C, N, O and Co in IL-Co@NC (**g**).

The prepared metal-containing ionic liquid was transferred to a tube furnace and annealed to 800 °C with a temperature ramp rate of 5 °C min^−1^ under a N_2_ environment. After holding at 800 °C for 1 h, it was allowed to cool down gradually. The calcination products were soaked in hydrochloric acid overnight and then washed repeatedly with deionized water and alcohol until neutral. The solid powders collected after grinding were designated as IL-Cu@NC and IL-Co@NC, respectively. The corresponding synthetic scheme is illustrated in Figure 1a.

### 2.3. Characterization

Detailed information on material characterization is available in the Supplementary Materials (Section S1).

## 3. Results

### 3.1. Characterization of Morphology and Structure

The microstructure and morphology of the as-prepared IL-Cu@NC and IL-Co@NC were investigated using SEM (Hitachi Ltd., Tokyo, Japan). As presented in Figure 1b, the surface of IL-Cu@NC appears dense with no discernible pore structure. At a scale of 200 nm (Figure 1c), the sample presents a rough and wrinkled morphology, further confirming the absence of obvious pore structures on its surface. Elemental mapping results in Figure 1d demonstrate the even dispersal of C, N, and O across the carbon matrix, along with the presence of the Cu element, which further confirms the successful loading of Cu. At the same magnification, compared with IL-Cu@NC, the IL-Co@NC exhibits pronounced porosity and an interconnected three-dimensional porous structure. Specifically, observation at low magnification (Figure 1e) demonstrates that the sample features a loose and porous structure, while the high-magnification image in Figure 1f further confirms that these pore walls are interconnected, forming a network-like structure. The EDS mapping results in Figure 1g confirm the homogeneous dispersion of N, O, and Co within the carbon framework.

**Figure 2:**
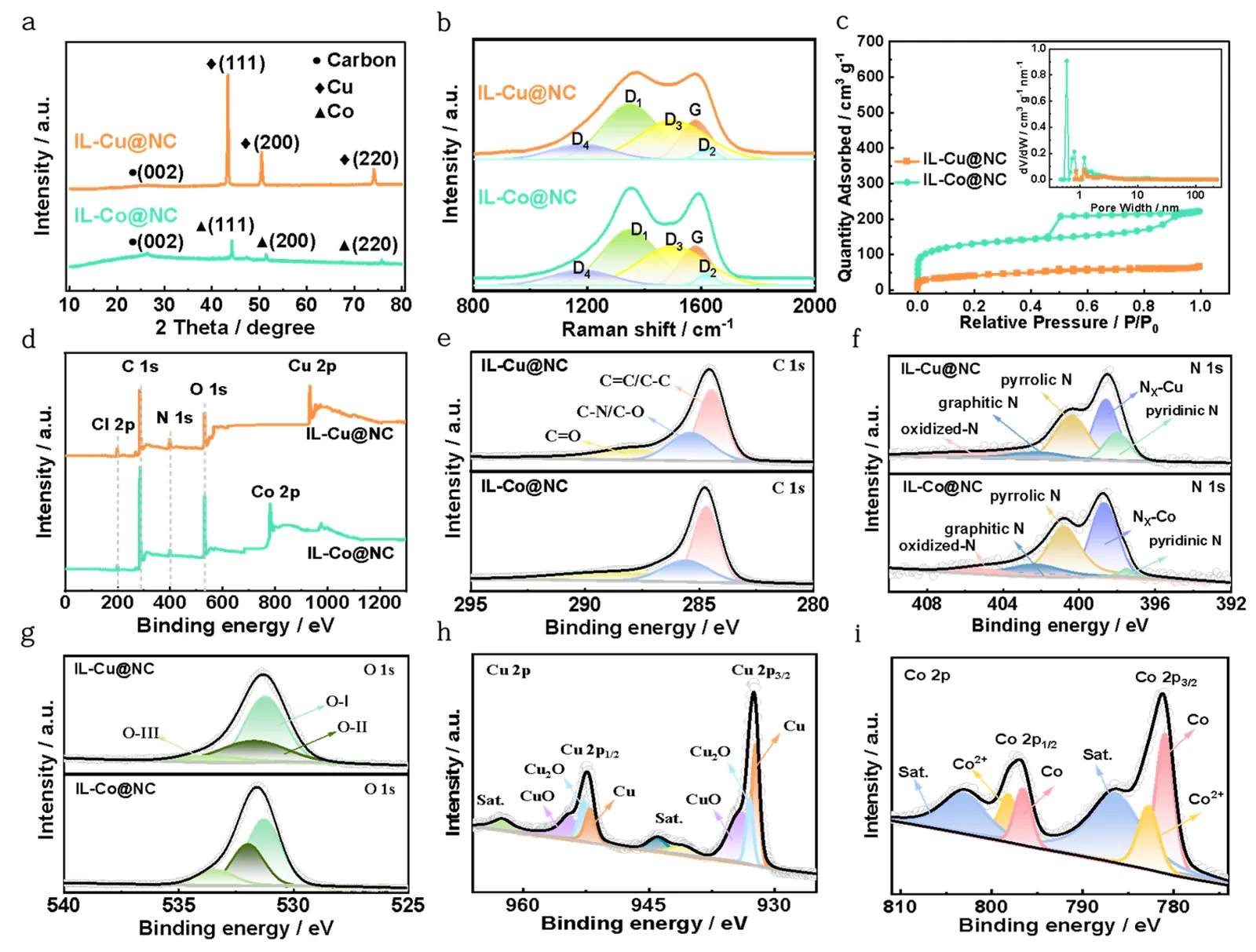
(**a**) XRD patterns; (**b**) Raman spectra; (**c**) N_2_ adsorption–desorption isotherms and pore size distributions of IL-Cu@NC and IL-Co@NC; The full XPS spectra of IL-Cu@NC and IL-Co@NC (**d**); the high-resolution spectra of C 1s (**e**); N 1s (**f**); O 1s (**g**); Cu 2p in IL-Cu@NC (**h**) and Co 2p in IL-Co@NC (**i**).

XRD (Shimadzu Corporation, Kyoto, Japan) analysis was carried out to elucidate the structural features of the as-prepared IL-Cu@NC and IL-Co@NC materials. As depicted in Figure 2a, it exhibits a broad diffraction peak at 26.5°, corresponding to the (002) crystal plane of graphitic carbon, indicating relatively low crystallinity. Furthermore, three distinct diffraction peaks can be observed at 43.5°, 50.4°, and 74.2°, corresponding to the (111), (200), and (220) crystal planes of Cu, respectively. This confirms the presence of zero-valent copper nanoparticles in IL-Cu@NC, promoting the kinetics of surface redox couples, thereby boosting the specific capacitance of the material [21,22]. As for the IL-Co@NC material, in addition to the (002) crystal plane of graphitic carbon, the XRD pattern also displays three diffraction peaks at 44.2°, 51.5°, and 75.6°, corresponding to the (111), (200), and (220) crystal planes of the metallic cobalt structure, respectively [23,24].

Raman (HORIBA Scientific, Longjumeau France) measurements were employed to assess the carbonization degree of the two samples, with the results displayed in Figure 2b. Two characteristic peaks emerge at 1359 cm^−1^ and 1586 cm^−1^, indexed to the D band and G band, respectively. To gain more detailed structural information, the Raman spectra were further deconvoluted following the Sadezky model [25], and the fitting results are summarized in Table S1. The G band was fitted with a Lorentzian function, while the D band was resolved into four components: D_1_ (~1350 cm^−1^, edge defects), D_2_ (~1620 cm^−1^, surface-disordered graphitic layers), D_3_ (~1500 cm^−1^, amorphous carbon), and D_4_ (~1180 cm^−1^, polyene-like structures). The area ratios reveal that IL-Cu@NC exhibits an I(D_3_)/I(G) ratio of 2.26, substantially higher than that of IL-Co@NC (1.02), indicating that the copper-derived carbon contains more amorphous carbon domains. Such amorphous regions are rich in structural disorder and edge sites, which can facilitate K^+^ ion adsorption at the electrode interface [26,27]. Additionally, IL-Cu@NC shows a higher I(D_4_)/I(G) ratio (0.80 vs. 0.51), suggesting more polyene-like structures or ionic impurities that may further contribute to pseudocapacitance. In contrast, IL-Co@NC exhibits higher I(D_1_)/I(G) (3.09 vs. 2.60) and I(D_2_)/I(G) (0.67 vs. 0.19) ratios, indicating more edge and surface defects, but its lower amorphous carbon content likely limits the availability of active sites.

To systematically investigate the specific surface area and pore structure characteristics of the as-prepared IL-Cu@NC and IL-Co@NC materials, N_2_ adsorption–desorption measurements (Micromeritics Instrument Corp., Norcross, GA, USA) were conducted on two samples. As revealed by Figure 2c, the adsorption isotherms of both samples exhibit type IV behavior, which is a typical feature of a hierarchical porous structure [28]. A sharp rising curve at low relative pressures indicates that micropores are predominant. A distinct hysteresis feature accompanying the gentle slope of the isotherm confirms the mesoporous architecture of the samples. The presence of minor macropores is corroborated by the tendency of the adsorption curve to plateau at high relative pressures. Although the isotherm types of the two samples are generally similar, there are differences in the shape of their hysteresis loops. The hysteresis loop of IL-Cu@NC is classified as type H3, while that of IL-Co@NC is of type H1. Furthermore, the IL-Cu@NC material displays a low degree of isothermal rise and no distinct hysteresis loop, indicating a less developed pore structure and smaller specific surface area. The corresponding pore size distribution shown in Figure 2c also confirms that IL-Co@NC possesses a hierarchical porous structure. The detailed structural parameters of the prepared IL-Cu@NC and IL-Co@NC samples are presented in Table S2. The specific surface area of IL-Co@NC (477.8 m^2^ g^−1^) is remarkably higher than that of IL-Cu@NC (143.9 m^2^ g^−1^), and the porosity of the IL-Co@NC sample is also significantly higher than that of IL-Cu@NC, indicating that the structural properties of the final material are highly contingent upon the choice of metal type [29,30]. These findings are consistent with the previous SEM observations.

XPS (Thermo Fisher Scientific, Waltham, MA, USA) was conducted to assess the chemical composition and bonding configurations of the prepared IL-Cu@NC and IL-Co@NC. As presented in Figure 2d and Table S3, the full XPS spectrum of the IL-Cu@NC sample reveals five characteristic peaks: Cl 2p (198.4 eV) [31], C 1s (284.8 eV) [32], N 1s (398.7 eV) [33], O 1s (532.9 eV) [34], and Cu 2p (932.6 eV) [35,36]. Similarly, the IL-Co@NC sample also exhibits Cl 2p (198.4 eV), C 1s (284.8 eV), N 1s (398.7 eV), O 1s (532.9 eV), and Co 2p (780.1 eV). This indicates that IL-Cu@NC contains C, O, N, Cl, and Cu, while IL-Co@NC possesses C, O, N, Cl, and Co. The high-resolution C 1s spectra of both samples (Figure 2e and Table S4) are deconvoluted into three peaks centered at 284.8 eV, 286.2 eV, and 288.5 eV, corresponding to C-C/C=C bonds, C-N/C-O bonds, and C=O bonds, respectively [37]. The existence of the C-N bond serves as definitive evidence for the successful incorporation of nitrogen atoms into the carbon structure. The N 1s spectra of IL-Cu@NC and IL-Co@NC (Figure 2f and Table S5) are fitted with five peaks attributed to pyridinic N (398.6 eV), Cu-N_X_/Co-N_X_ coordination (399.3 eV) [38,39], pyrrolic N (401.1 eV), graphitic N (402.6 eV), and oxidized N (404.5 eV). The presence of graphitic N in the carbon framework is conducive to improving electrical conductivity, and the overall nitrogen-containing functional groups collectively contribute to enhanced electrochemical performance [6,16,40]. The O 1s spectra (Figure 2g and Table S6) can be resolved into three peaks at 531.5 eV (O-I), 532.0 eV (O-II), and 533.4 eV (O-III), corresponding to oxygen in metal oxides, the C=O group, and the C-O group, respectively. The appearance of the O-I peak directly confirms that transition metals (Cu/Co) are present in the form of oxides or coordination compounds. Significantly, the oxygen-containing functional groups corresponding to O-II and O-III can enhance the surface hydrophilicity of carbon materials and provide additional active sites for pseudocapacitive reactions [38]. To further elucidate the chemical state of the copper species in IL-Cu@NC and the effect on electrochemical performance, high-resolution XPS spectra of Cu 2p were analyzed. As presented in Figure 2h, it exhibits a characteristic bimodal structure resulting from spin–orbit coupling. After deconvolution, three component peaks at 932.5, 933.3, and 935.0 eV can be resolved in the Cu 2p_3/2_, while three component peaks located at 952.4, 953.7, and approximately 955.0 eV appear in the Cu 2p_1/2_. These two energy regions exhibited good spin–orbit correspondence, indicating that copper in the material exists in three different chemical states [36,41,42]. Specifically, the double peak at 932.5/952.4 eV is attributed to metallic Cu^0^ [43], while the double peak at 933.3/953.7 eV is associated with Cu^+^ in Cu_2_O, and the double peak at 935.0 eV and 955.0 eV is attributed to Cu^2+^ in CuO. The latter features strong satellite peaks at binding energies of 941.9 eV, 944.4 eV, and 962.8 eV, which are typical signals of Cu^2+^, further confirming the presence of the CuO species. Furthermore, according to the quantitative assessment of the high-resolution Cu 2p XPS spectrum (Tables S3 and S7), the total copper content is 6.0 at%, with relative contents of Cu^0^, Cu_2_O, and CuO at 46.3%, 23.7%, and 30.0%, respectively. Remarkably, the presence of Cu^2+^ species and satellite peaks indicates that the surface of IL-Cu@NC exposes abundant active copper sites in various oxidation states, while the presence of Cu^0^ provides a low-resistance pathway for electron transport. The synergistic coexistence of these multivalent copper species facilitates efficient charge transfer and thorough Faradaic reactions in IL-Cu@NC during charging and discharging, thereby enhancing its capacitive performance [44]. As for the Co 2p spectrum (Figure 2i and Table S7), it exhibits a doublet consisting of 2p_3/2_ and 2p_1/2_ peaks caused by spin–orbit coupling. The Co 2p_3/2_ spectrum can be fitted to two characteristic peaks at 779.5 eV and 782.1 eV, respectively, while the corresponding Co 2p_1/2_ peak appears at 797.2 eV and 798.9 eV. The two peaks at 779.5/797.2 eV are attributed to the metallic Co^0^ state, while another two peaks at 782.1/798.9 eV correspond to Co^2+^, which acts as a pseudocapacitive active site and contributes to Faradaic energy storage. Meanwhile, the satellite peaks at 787 eV and 803.4 eV further confirm the presence of Co^2+^ [45,46,47,48].

### 3.2. Characterization of the Electrochemical Properties of Materials

**Figure 3:**
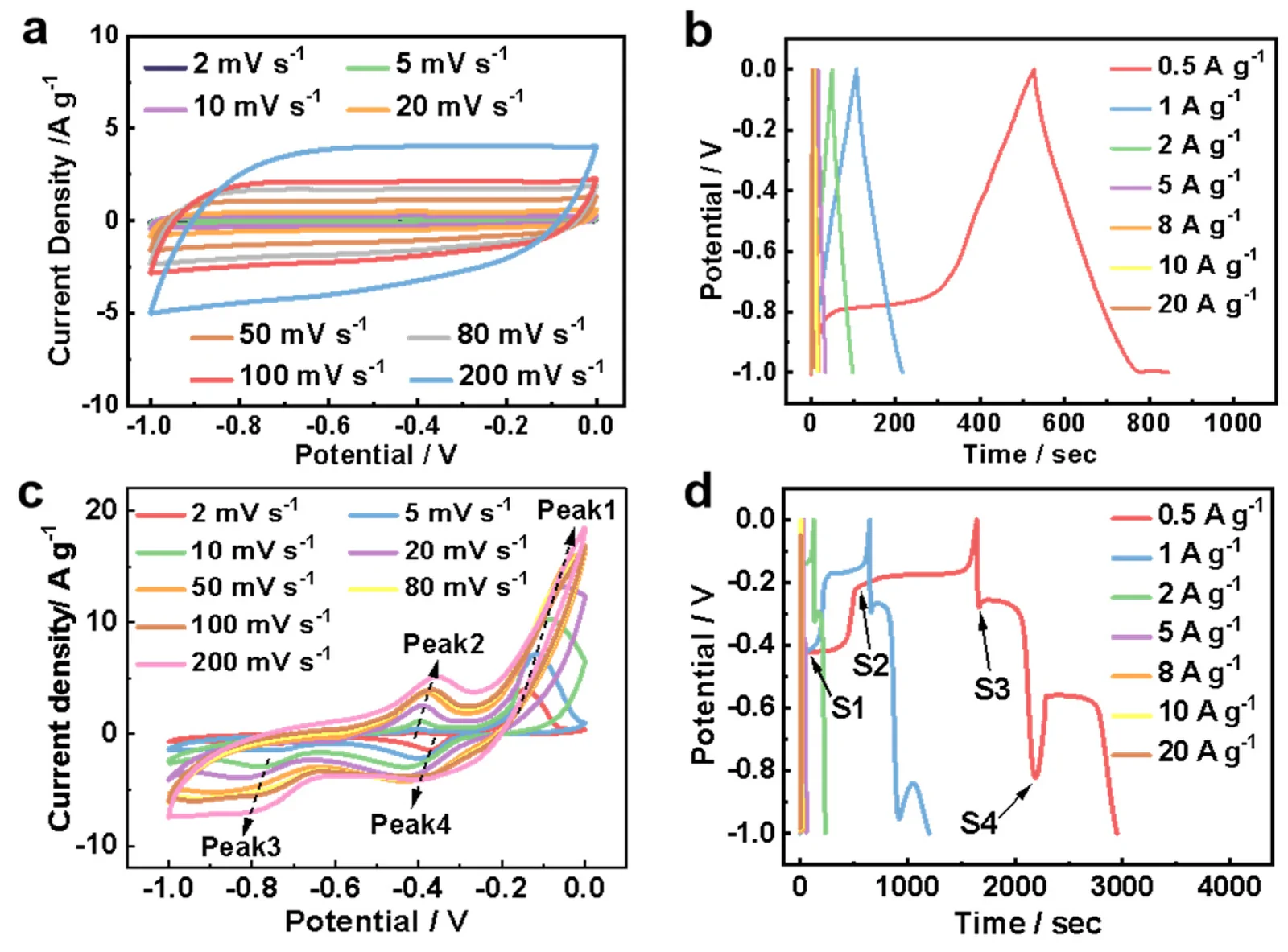
CV profiles at various scan rates of IL-Co@NC (**a**) and IL-Cu@NC (**c**); GCD curves over different current rates of IL-Co@NC (**b**) and IL-Cu@NC (**d**).

Using a three-electrode configuration (Shanghai Chenhua Instrument Co., Ltd., Shanghai, China), the electrochemical performance of IL-Cu@NC and IL-Co@NC was evaluated in a 6 M KOH solution; the experimental parameters and calculation formulas are listed in Section S2 of the Supplementary Materials. The electrochemical energy storage characteristics of IL-Cu@NC and IL-Co@NC were investigated through cyclic voltammetry (CV) curves. Figure 3a reveals that the CV profile of IL-Co@NC displays a near-rectangular shape, which remains well preserved with increasing scan rates, indicating that this material possesses superior capacitive behavior of the electrical double layer and rapid current response [49]. When the scan rate is raised to as high as 200 mV s^−1^, the CV curve still maintains well-defined symmetry and shows no significant polarization distortion, fully confirming that IL-Co@NC possesses excellent rate capability, which is attributed to the efficient ion transport pathways formed by its three-dimensional interconnected carbon framework [50]. However, the CV curves of IL-Cu@NC (Figure 3c) exhibit an asymmetric shape at different scan rates, suggesting that the electrochemical energy storage primarily stems from Faradaic pseudocapacitance [51,52]. Specifically, four distinct redox peaks are observed in the CV curves of IL-Cu@NC, which arise from the coexistence of multiple copper species with different valence states on the nitrogen-doped carbon matrix. The oxidation peaks, designated as Peak 1 and Peak 2, appear at approximately −0.12 V and −0.39 V, while the reduction peaks, Peak 3 and Peak 4, appear at approximately −0.82 V and −0.38 V, respectively. According to the XPS results, the copper species in IL-Cu@NC exist in three valence states, namely Cu^0^, Cu^+^ in Cu_2_O, and Cu^2+^ in CuO. Accordingly, Peak 1 corresponds to the oxidation of Cu^0^ to Cu^+^, and Peak 2 corresponds to the further oxidation of Cu^+^ to Cu^2+^. On the reverse scan, Peak 3 is attributed to the reduction of Cu^2+^ to Cu^+^, while Peak 4 corresponds to the subsequent reduction of Cu^+^ to Cu^0^. As the scan rate increases from 2 mV s^−1^ to 200 mV s^−1^, the separation between the oxidation and reduction peaks gradually increases, indicating that these redox processes are quasi-reversible in nature [53,54,55]. These results suggest that the pseudocapacitive behavior of IL-Cu@NC originates from the stepwise redox transitions between Cu^0^ and Cu^2+^. Additionally, the galvanostatic charge–discharge (GCD) curve serves as an essential benchmark for evaluating supercapacitor performance. The shape of the GCD curve can intuitively reflect the charge–discharge reversibility and electrochemical stability of the electrode materials. To further verify the results of the CV analysis, constant-current charge–discharge measurements were performed on the IL-Cu@NC and IL-Co@NC samples at different current densities. The corresponding data are shown in Figure 3b. The IL-Co@NC samples generally present an isosceles triangle shape, indicating high electrochemical reversibility. Notably, the GCD curve of IL-Co@NC exhibits slight charging and discharging plateaus at 0.5 A g^−1^, which is primarily attributed to the conversion of Co^2+^ and Co0 within the electrode at low current density [56,57]. Moreover, a specific capacitance of 158.57 F g^−1^ is derived from the GCD curve at 0.5 A g^−1^. Obviously, IL-Cu@NC exhibits a longer discharge time at the same current density, exhibiting a specific capacitance of 653 F g^−1^ (Figure 3d), which is significantly greater than that of the IL-Co@NC material. Meanwhile, compared to IL-Co@NC, the GCD curves of IL-Cu@NC exhibit distinct asymmetry and characteristic voltage plateaus, revealing that the charge storage kinetics are primarily attributed to Faradaic pseudocapacitive contributions. As observed in the CV test, the charge–discharge plateaus match well with the redox peaks in terms of potential. Specifically, the slope changes indicated by arrows S1, S2, S3, and S4 in the GCD curves (Figure 3d) reflect the stepwise redox transitions of copper species during charge and discharge. S1 marks the onset of the first charge plateau, corresponding to the oxidation of Cu^0^ to Cu^+^. S2 marks the onset of the second charge plateau, corresponding to the further oxidation of Cu^+^ to Cu^2+^. On the discharge side, S3 marks the onset of the discharge plateau, corresponding to the reduction of Cu^2+^ to Cu^+^. S4 marks the sharp drop following the discharge plateau, where the reaction transitions from charge-transfer control to diffusion-limited control as the electroactive species near the electrode surface become depleted, after which the reduction of Cu^+^ to Cu^0^ occurs. These plateaus are ascribed to the electrochemical redox reactions of copper species (Cu^0^ ↔ Cu^+^ ↔ Cu^2+^) [58,59]. However, upon raising the current density from 0.5 to 20 A g^−1^, the charge–discharge time significantly shortened, and the voltage plateaus gradually weakened, reflecting the constraints on rate performance imposed by diffusion-controlled processes in Cu-based materials [60,61,62,63].

**Figure 4:**
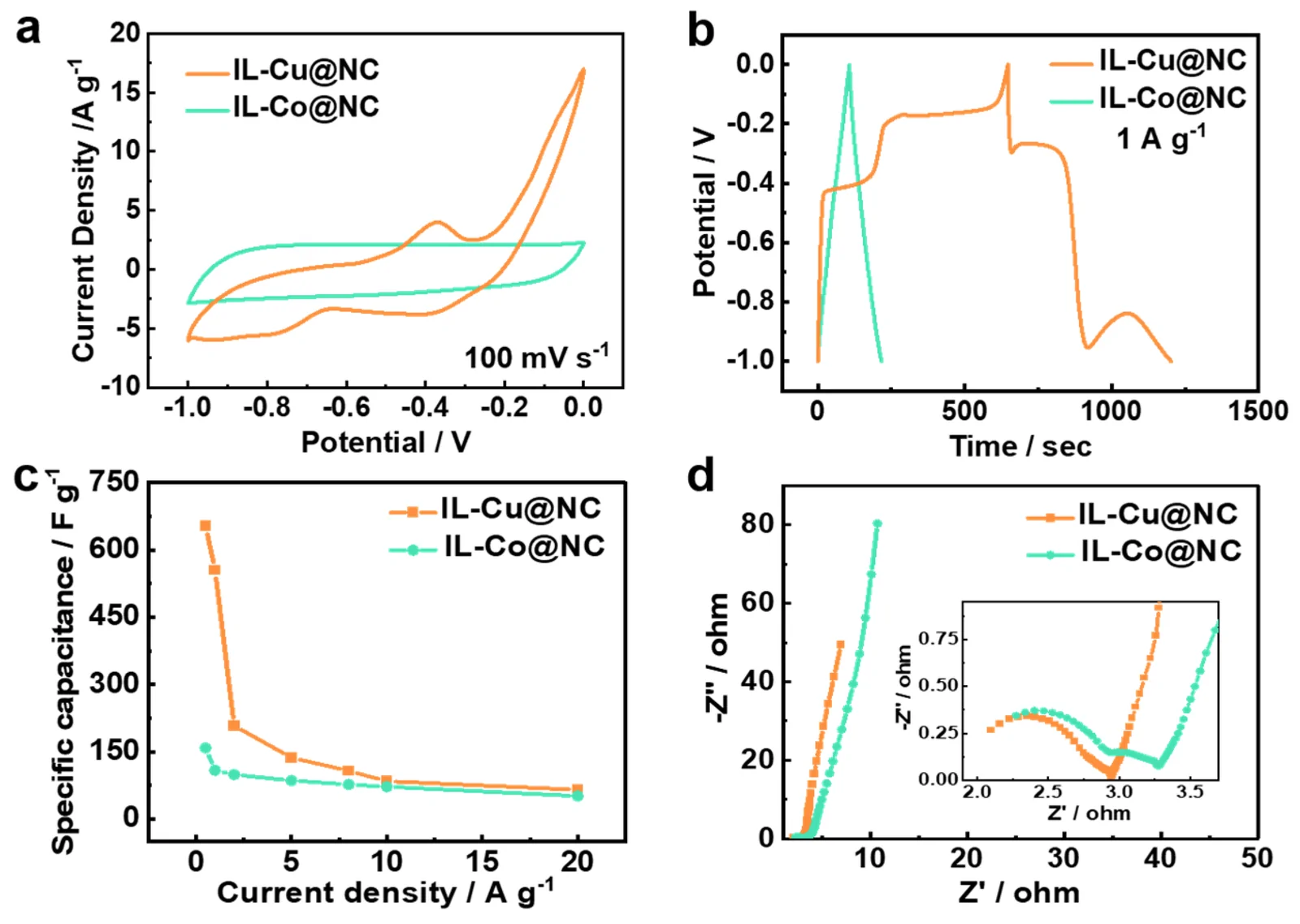
(**a**) CV curves recorded at 100 mV s^−1^; (**b**) GCD plots at 1 A g^−1^; (**c**) dependence of specific capacitance on current density; (**d**) Nyquist plot of IL-Cu@NC and IL-Co@NC.

The CV comparison results in Figure 4a further confirm the significant advantages of IL-Cu@NC concerning electrochemical energy storage performance. At the scan rate of 100 mV s^−1^, the current density response of IL-Cu@NC surpasses that of IL-Co@NC; meanwhile, the CV curve of IL-Cu@NC encloses a larger area than that of IL-Co@NC. Furthermore, IL-Cu@NC also exhibits a longer discharge time at the same current density, as shown in Figure 4b. These results collectively indicate that the IL-Cu@NC material possesses a higher specific capacitance and superior charge storage capacity. Figure 4c illustrates the GCD measurement results for IL-Cu@NC and IL-Co@NC at different current densities. It is evident that the capacitance of IL-Cu@NC is superior to that of IL-Co@NC, demonstrating the excellent capacitive performance and favorable rate response. To further evaluate the charge transfer kinetics of the IL-Cu@NC and IL-Co@NC electrodes, electrochemical impedance spectroscopy tests were conducted. Figure S2 provides the corresponding equivalent circuits, with the fitted parameters summarized in Table S8. The Nyquist plots of both materials (Figure 4d) exhibit a semicircular arc in the high-frequency range and a straight-line behavior in the low-frequency range. Notably, the diameter of the semicircular arc for IL-Cu@NC is much smaller than that of IL-Co@NC, indicating a more efficient interfacial charge transfer process [64]. The R_ct_ of IL-Cu@NC is 1.17 Ω, obtained from the equivalent circuit fitting, which is lower than the charge transfer impedance (3.39 Ω) of IL-Co@NC, indicating that the IL-Cu@NC material exhibits faster Faradaic reaction kinetics at the electrode/electrolyte interface. This result is consistent with the higher specific capacitance exhibited by IL-Cu@NC in the CV and GCD measurements.

**Figure 5:**
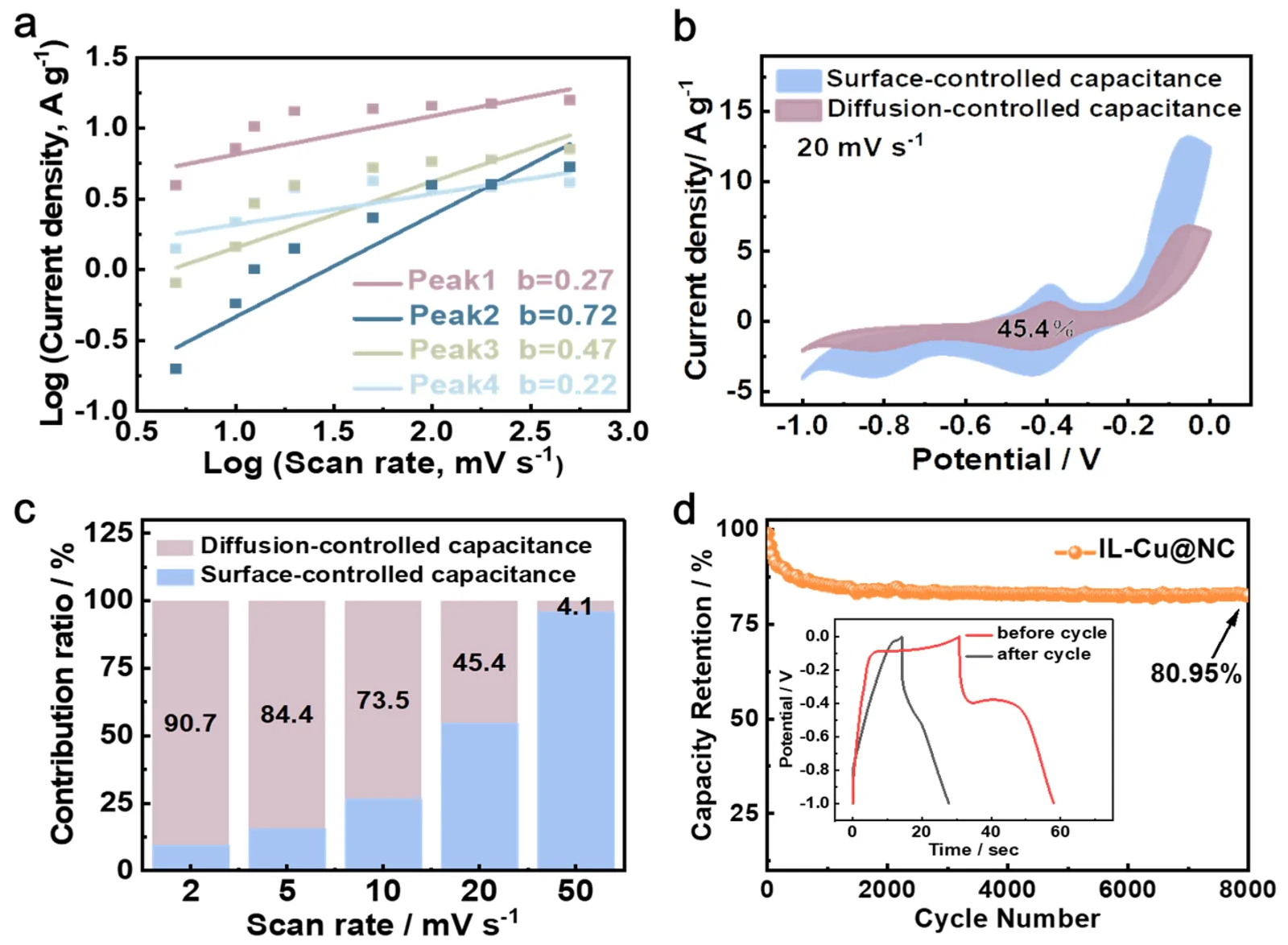
(**a**) Calculated b values of IL-Cu@NC; (**b**) contribution of surface-controlled and diffusion-controlled processes at 20 mV s^−1^; (**c**) capacitive contribution as a function of scan rate; (**d**) cycle stability of IL-Cu@NC at 5 A g^−1^.

To further investigate the charge storage behavior of the IL-Cu@NC electrode, the b values for each redox peak were calculated using the formula in Section S3, based on the CV data obtained at different scan rates. As shown in Figure 5a, the b values for IL-Cu@NC range from 0.22 to 0.72, with most values approaching 0.5. This indicates that the charge storage is predominantly governed by diffusion-controlled Faradaic processes at lower scan rates [65]. Such behavior is attributed to the copper species embedded in the nitrogen-doped carbon matrix, which undergo stepwise redox transitions involving ion diffusion into the bulk of the material, consistent with the XPS results and the redox peak assignments discussed above. As presented in Figure 5b, the diffusion-controlled contribution at 20 mV s^−1^ is 45.4%. Figure 5c provides a quantitative comparison of the surface-controlled and diffusion-controlled contributions at different scan rates. As the scan rate increases from 2 to 50 mV s^−1^, the diffusion-controlled proportion gradually decreases from 90.7% to 4.1%, while the surface-controlled contribution increases correspondingly. This transition occurs because at higher scan rates, the time available for ion diffusion into the bulk is limited, shifting the charge storage toward surface-confined reactions [66,67]. These results indicate that the IL-Cu@NC electrode exhibits a mixed charge storage mechanism. At low scan rates, the charge storage is dominated by diffusion-controlled Faradaic reactions, whereas at high scan rates, surface-controlled capacitive contributions become increasingly significant. This dual behavior is consistent with the characteristic charge-discharge plateaus observed in the GCD profiles and the asymmetric redox peaks in the CV curves. As demonstrated in Figure 5d, the prepared IL-Cu@NC electrode maintains 80.95% of its initial capacitance following 8000 cycles under 5 A g^−1^, indicating stable long-term cycling stability under the tested conditions.

To further evaluate the electrochemical performance of IL-Cu@NC, its specific capacitance obtained from three-electrode measurements was compared with those of previously reported metal-loaded carbon-based composites and nitrogen-doped carbon materials as summarized in Table S9 [68,69,70,71,72,73,74,75,76,77]. The results show that the specific capacitance of IL-Cu@NC is higher than that of most comparable materials in three-electrode measurements, indicating its favorable charge storage capability as an electrode material. The electrochemical performance is attributed to several factors: (1) The defect sites introduced by N doping effectively enhance the electrochemically active area, which favors the contribution of double-layer capacitance. (2) The introduction of metal copper significantly enhances the Faradaic pseudocapacitance contribution; in particular, the reversible valence transition between Cu^0^ and Cu^2+^ provides abundant redox-active sites, contributing substantial pseudocapacitance during charging and discharging. (3) The Cu-N_X_ coordination bond structure acts as an efficient charge-transfer bridge, facilitating interfacial electron transfer and significantly improving the kinetics of Faradaic reactions.

## 4. Conclusions

Employing the prepared metal ionic liquids ([BMIm]_2_[CuCl_4_] and [BMIm]_2_[CoCl_4_]) as both carbon precursors and metal sources, metal-loaded carbon composite materials have been directly synthesized via high-temperature carbonization. Systematic investigations were implemented to probe the effects of different metal anions on the electrochemical performance of the carbon materials. Electrochemical studies demonstrate that the prepared IL-Cu@NC electrode exhibits a specific capacitance of 653 F g^−1^ at 0.5 A g^−1^, which is significantly higher than the 158.6 F g^−1^ of IL-Co@NC, and it retains 80.95% of its initial capacitance over 8000 cycles, indicating stable cycling performance. The exceptional energy storage performance arises from the synergistic interaction: the double-layer capacitance contributed by N-doping, the rapid Faradaic reactions facilitated by multivalent copper species, and the abundant defect sites introduced by the Cu-N_X_ coordination structure. This work provides experimental substantiation for the implementation of metal-ion-liquid-derived carbon materials within the domain of electrochemical energy storage and offers a feasible approach for the controlled design of high-performance supercapacitor electrode materials.

## Data Availability

The original contributions presented in this study are included in the article/Supplementary Material. Further inquiries can be directed to the corresponding author.

## Supplementary Materials

The following supporting information can be downloaded at https://www.mdpi.com/article/10.3390/ma19173687/s1, Section S1: Materials characterization; Section S2: Electrochemical performance evaluation; Section S3: Charge Storage Mechanisms in Supercapacitors, Capacitive Contributions, and Related Formulas; Figure S1: The synthetic route of metal-containing ionic liquid; Figure S2: The equivalent circuit diagram of the prepared electrode; Table S1: Raman fitting area ratios for IL-Cu@NC and IL-Co@NC; Table S2: Pore volume and specific surface area parameters of carbon materials derived from metal-containing ionic liquids; Table S3: Surface elemental content of the carbon materials from XPS measurements; Table S4: Relative atomic percentages of C 1s species in IL-Cu@NC and IL-Co@NC; Table S5: Relative atomic percentages of N 1s species in IL-Cu@NC and IL-Co@NC; Table S6: Relative atomic percentages of O 1s species in IL-Cu@NC and IL-Co@NC; Table S7: Relative contents of Cu species in IL-Cu@NC and Co species in IL-Co@NC; Table S8: Simulated parameters of the elements of EIS for samples; Table S9: Comparison of electrochemical performance of IL-Cu@NC electrode and Cu-doped carbon electrode. Refs. [68,69,70,71,72,73,74,75,76,77] are included in Supplementary Materials.
