# Supplementary material for: Nitrogen-Doped Carbon Composites Embedded with Cu/Co Species Derived from Metal-Functionalized Ionic Liquids: Design and Supercapacitive Performance
Source: Materials (Basel). 2026 Aug 30;19(17):3687. doi: 10.3390/ma19173687 (PMC13567657; doi:10.3390/ma19173687)
Supplement: Supplementary file 1 [file materials-19-03687-s001.zip › materials-4472796-supplementary.pdf]

materials

MDPI

Supplementary Material

Nitrogen-Doped Carbon Composites Embedded with Cu/Co Species Derived from Metal-Functionalized Ionic Liquids: Design and Supercapacitive Performance

Jiao Wu, Lingxia Liu, Liu Liu, Zhenning Zhuo, Zefeng Wei, Zhi Cui, Congxiu Guo and Jiangtao Meng *

- School of Electric Power, Civil Engineering and Architecture, Shanxi University, Taiyuan 030006, China; jiaow@sxu.edu.cn (J.W.); liulingxia@sxu.edu.cn (L.L.); liuliu1@sxu.edu.cn (L.L.)
- * Correspondence: mjiangtao@sxu.edu.cn

Section S1. Materials characterization

The microstructure and morphological characteristics of the samples were observed using a scanning electron microscope (SEM, SU8010-Hitachi) equipped with an energy dispersive spectrometer (EDS) and a transmission electron microscope (TEM, HT-7700). X-ray diffraction (XRD) patterns were obtained on a Labx XRD-6000 instrument (Shimadzu, Japan) with Cu Kα radiation (wavelength = 0.15418 nm) and a scan range of 2θ = 10-80° (scan speed 2° min^{-1}) at room temperature. Internal defects and the degree of graphitization of material were analyzed through Raman spectroscopy (Raman, XploRA PLUS, HORIBA, France). Nitrogen adsorption-desorption experiments were conducted on an ASAP 2460 analyzer at 77 K, with the specific surface area (SSA) calculated using the BET method and the pore size distribution based on density functional theory (DFT). The elemental composition and the chemical states of heteroatoms in samples were precisely analyzed using X-ray photoelectron spectroscopy (XPS, Escalab 250Xi) technology.

Section S2.electrochemical performance evaluation

In this study, a three-electrode system was used to test the electrochemical performance of the electrode materials, with a 6 M KOH aqueous solution serving as the electrolyte. The three-electrode test system, in conjunction with an electrochemical workstation, was used to perform cyclic voltammetry, constant-current charging and discharging, and electrochemical impedance spectroscopy (EIS) tests.

The GCD curves were measured at a constant current density of 1 A g^{-1}, and the corresponding specific capacitance was calculated using Equation (S1).

To investigate the electrochemical properties, cyclic voltammetry curves were recorded at different scan rates (2 mV s^{-1}, 5 mV s^{-1}, 10 mV s^{-1}, 20 mV s^{-1}, 50 mV s^{-1}, 100 mV s^{-1}, 200 mV s^{-1}).

To test the electrochemical stability of the relevant materials, GCD curves were obtained at current densities ranging from 0.5 to 20 A g^{-1} (0.5 A g^{-1}, 1 A g^{-1}, 2 A g^{-1}, 5 A g^{-1}, 8 A g^{-1}, 10 A g^{-1}, 20 A g^{-1}), the specific capacitance values were calculated using Equation (S1).

EIS testing, also known as impedance spectroscopy testing, is one of the most common testing methods in the field of electrochemistry, with EIS testing conducted over a wide frequency range of 0.01-100 kHz using a 5 mV signal amplitude.

:

$$C = \frac{I \triangle t}{m \triangle V} \quad \text{(S1)}$$

V

oltage window (V); I: Discharge current (A); m: Mass of active material in the electrode (g); Δ t-discharge time (s).

Section S3. Charge Storage Mechanisms in Supercapacitors, Capacitive Contributions, and Related Formulas

To comprehensively investigate the effect of metal ionic liquids as carbon precursors on capacitive performance, the charge-discharge kinetics of the prepared carbon
e
l

$$i = av^b \quad \text{(S2)}$$

e

$$log(i) = b\ log(v) + log(a) \quad \text{(S3)}$$

ctrodes were also evaluated using following Equations (S2) and (S3)

where i is the current density, v represents the scan rate, and the b value can directly reflect the reaction kinetics of the electrodes, with the value close to 0.5 implying the diffusion-controlled behavior, while close to 1 indicating the surface-controlled process.

The capacitance contributions from surface-controlled processes and diffusion-controlled processes are investigated according to the following Equation (S4) and (S5):

$$i = k_1 v + k_2 v^{1/2} \quad \text{(S4)}$$

$$i/v^{1/2} = k_1 v^{1/2} + k_2 \quad \text{(S5)}$$

where $k_1 v$ and $k_2 v^{1/2}$ signify the surface-controlled as well as the diffusion-controlled processes, respectively.

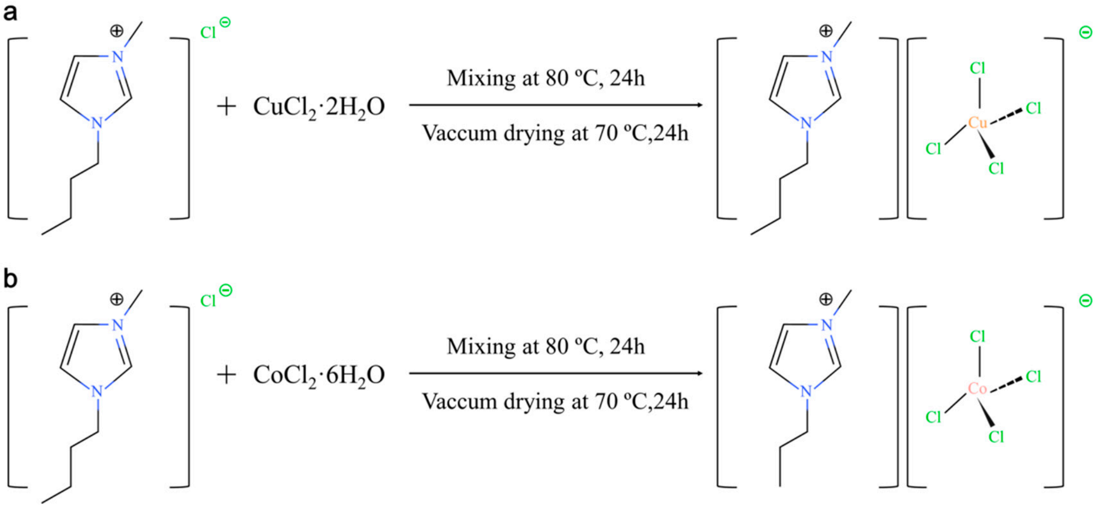

Figure S1. The synthetic route of metal-containing ionic liquid.

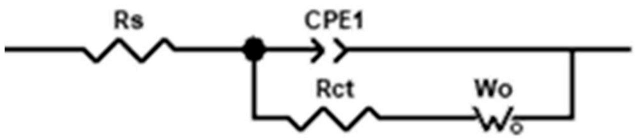

Figure S2. The equivalent circuit diagram of the prepared electrode.

Table S1. Raman fitting area ratios for IL-Cu@NC and IL-Co@NC

Sample	I(D1)/I(G)	I(D3)/I(G)	I(D2)/I(G)	I(D4)/I(G)
IL-Cu@NC	2.60	2.26	0.19	0.80

IL-Co@NC	3.09	1.02	0.67	0.51

Table S2. Pore volume and specific surface area parameters of carbon materials derived from metal-containing ionic liquids.

Samples	S_{BET} (m^2 g^{-1})	S_{mic} (m^2 g^{-1})	V_{total} (cm^3 g^{-1})	V_{mic} (cm^3 g^{-1})	Average pore size (nm)
IL-Cu@NC	143.9	18.6	0.10	0.008	2.82
IL-Co@NC	477.8	359.1	0.34	0.154	2.83

Table S3. Surface elemental content of the carbon materials from XPS measurements.

Sample	C (at. %)	N (at. %)	O (at. %)	Cl (at. %)	Cu (at. %)	Co (at. %)
IL-Cu@NC	66.0	8.7	15.8	3.5	6.0	-
IL-Co@NC	69.5	4.6	17.9	1.6	-	6.4

Table S4. Relative atomic percentages of C 1s species in IL-Cu@NC and IL-Co@NC.

Samples	C-C/C=C 284.8(eV)	C-N/C-O 286.2(eV)	C=O 288.5(eV)
IL-Cu@NC(wt%)	61.96	24.53	13.51
IL-Co@NC(wt%)	64.72	26.17	9.13

Table S5. Relative atomic percentages of N 1s species in IL-Cu@NC and IL-Co@NC.

Samples	Pyridinic N 398.6(eV)	Cu/Co-N_x 399.3(eV)	Pyrrolic N 401.1(eV)	Graphitic N 402.6(eV)	Oxidized N 404.5(eV)
IL-Cu@NC(wt%)	19.38	23.09	43.45	8.37	5.70
IL-Co@NC(wt%)	49.34	5.82	35.29	6.76	2.80

Table S6. Relative atomic percentages of O 1s species in IL-Cu@NC and IL-Co@NC.

Samples	O-I 531.5(eV)	O-II 532(eV)	O-III 533.4(eV)
IL-Cu@NC(wt%)	73.78	21.87	4.35

IL-Co@NC(wt%)	48.68	35.01	16.31

Table S7. Relative contents of Cu species in IL-Cu@NC and Co species in IL-Co@NC.

Samples	Cu^0 932.5/952.4 (eV)	$Cu^+(Cu_2O)$ 933.3/953.7 (eV)	$Cu^{2+}(CuO)$ 935.0/955.0 (eV)	Co^0 779.5/797.2 (eV)	Co^{2+} 782.1/798.9 (eV)
IL-Cu@NC(wt%)	46.3	23.7	30.0	-	-
IL-Co@NC(wt%)	-	-	-	64.60	35.40

Table S8. Simulated parameters of the elements of EIS for samples.

Samples	R_s	R_{ct}	CPE1	W_o
IL-Cu-800	1.58	1.17	0.38	0.22
IL-Co-800	1.72	3.39	0.65	0.42

Table S9. Comparison of electrochemical performance of IL-Cu@NC electrode and Cu-doped carbon electrode.

Samples	C ($F g^{-1}$)	SSA ($m^2 g^{-1}$)	Cycling stability	Number of Cycles	E ($Wh kg^{-1}$)	P ($W kg^{-1}$)	Ref.
Fe-OHPC	283 (0.5 $A g^{-1}$)	1638	99.8% (5 $A g^{-1}$)	10000	16.5	300	[1]
Zn-C	285 (1 $A g^{-1}$)	1464	94.5% (50 $A g^{-1}$)	10000	13.7	650	[2]
Co-NC	310 (0.5 $A g^{-1}$)	206	87% (5 $A g^{-1}$)	5000	59.73	15751	[3]
CCAC-Fe	338 (1 $A g^{-1}$)	2305.7	97.5% (10 $A g^{-1}$)	5000	18.6	455	[4]

Fe-NC	293.6 (0.5 A g-1)	567	92.6% (10 A g-1)	10000	8.68	137.53	[5]
N-CA-Cu	508.5 (0.5 A g-1)	1987.76	95.1% (5 A g-1)	12000	12.56	125	[6]
HPC-RH 3/1	352 (1 A/g)	2214	100 % (10 A/g)	10000	6.98	500	[7]
PC-CNTs	224.4 (1 A/g)	2178.1	98 % (1 A/g)	20000	12.15	6.25	[8]
C-0.05N-700	344.3 (0.25 A/g)	2548	100 % (10 A/g)	20000	8.1	25.2	[9]
CHPC-900	335 (0.5 A/g)	1817	96.2 % (1 A/g)	20000	9.9	125	[10]
IL-Cu@NC	653 (0.5 A g-1)	143.89	80.95% (5 A g-1)	8000	-	-	This work

References

1. Liu, H.; Wang, Y.; Lv, L.; et al. Oxygen-enriched hierarchical porous carbons derived from lignite for high-performance supercapacitors. *Energy* **2023**, 269, 126707.
2. Liu, N.; Liu, X.; Pan, J. A new rapid synthesis of hexagonal prism Zn-MOF as a precursor at room temperature for energy storage through pre-ionization strategy. *Journal of Colloid and Interface Science* **2022**, 606, 1364-1373.
3. Devi, B.; Jain, A.; Roy, B.; et al. Cobalt-Embedded N-Doped Carbon Nanostructures for Oxygen Reduction and Supercapacitor Applications. *ACS Applied Nano Materials* **2020**, 3, 6354-6366.
4. Xu, Z.; Zhang, X.; Yang, X.; et al. Synthesis of Fe/N Co-doped Porous Carbon Spheres Derived from Corncob for Supercapacitors with High Performances. *Energy & Fuels* **2021**, 35, 14157-14168.
5. Fan, L.; Hou, Z.; Liu, T.; et al. Fe-N_x-C material with excellent supercapacitor performance prepared through oleic acid acidification pretreatment—Oily sludge pyrolysis process. *Journal of Environmental Chemical Engineering* **2022**, 10, 108256.
6. Zhang, Z.; Lu, S.; Li, Y.; et al. Promoting hierarchical porous carbon derived from Bamboo via copper doping for high-performance supercapacitors. *Industrial Crops and Products* **2023**, 203, 117155.
7. Zhang, X.; Sun, B.; Fan, X.; Liang, P.; Zhao, G.; Saikia, B.K.; Wei, X. Hierarchical Porous Carbon Derived from Coal and Biomass for High Performance Supercapacitors. *Fuel* **2022**, 311, 122552. doi:10.1016 /j.fuel.2021.122552.
8. Zhao, R.; Hao, J.; Yang, X.; Zhao, Y.; Chen, J.; Guo, J.; Chi, C.; Guo, Q. $FeCl_3$ Induced Catalytic Activation Prepared Porous Carbon with Carbon Nanotube Skeleton from Coal for Supercapacitor Electrodes. *J. Power Sources* **2024**, 623, 235436. doi:10.1016/j.jpowsour.2024, 235436.
9. Chen, P.; Tang, Y.; Gao, Y.; Zhang, Y.; Liu, L. Design and Structure Optimization of Coal-Based Hierarchical Porous Carbon by Molten Salt Method for High-Performance Supercapacitors. *J. Power Sources* **2023**, 580, 233334. doi:10.1016/j.jpowsour.2023.233334.
10. Dong, D.; Xiao, Y.; Xing, J. Facile Wet Mechanochemistry Coupled K_2FeO_4 Activation to Prepare Functional Coal-Derived Hierarchical Porous Carbon for Supercapacitors. *J. Clean. Prod.* **2023**, 428, 139474. doi:10.1 016/j.jclepro.2023.139474
